# Adherence to General Medical Screenings, Cancer-Specific Screenings, and Management of Chronic Diseases in Cancer Survivors: Focus on Five-Year Survivors

**DOI:** 10.3390/cancers17142394

**Published:** 2025-07-19

**Authors:** EunKyo Kang, HyeWon Lee, Juyoung Choi, HyoRim Ju

**Affiliations:** 1 National Cancer Control Institute, National Cancer Center, Goyang 10408, Republic of Korea; 76756@ncc.re.kr (H.L.); cjy1222@ncc.re.kr (J.C.); 2 Department of Family Medicine, National Cancer Center, Goyang 10408, Republic of Korea; 3 Department of Family Medicine, Dankook University Hospital, Cheonan 31116, Republic of Korea; jjurim@naver.com; 4 Department of Family Medicine, Seoul National University College of Medicine, Seoul 03080, Republic of Korea

**Keywords:** cancer survivors, general medical screenings, cancer-specific screenings, chronic disease management

## Abstract

Background: Cancer survivors may continue to experience health issues that affect their quality of life and raise the risk of other chronic diseases. Methods: This study aimed to assess adherence to general health check-ups, cancer-specific screenings, and chronic disease management among five-year cancer survivors using nationally representative data from the Korea National Health and Nutrition Examination Survey (KNHANES) conducted between 2013 and 2021. A total of 2597 cancer survivors and 2458 matched non-cancer controls were selected via 1:1 propensity score matching based on demographic and lifestyle characteristics. We analyzed health behaviors (smoking and alcohol use), participation in general and cancer-specific screenings, and medication adherence for hypertension, diabetes, and dyslipidemia. Results: Compared to controls, cancer survivors, especially those diagnosed more than five years ago, were significantly less likely to participate in recommended cancer screenings (adjusted OR: 0.82, *p* = 0.014) and had lower adherence to treatment for hypertension (adjusted OR: 1.84, *p* = 0.004) and dyslipidemia (adjusted OR: 1.42, *p* = 0.026). However, cancer survivors were less likely to smoke or consume alcohol. Conclusions: These findings underscore the need for comprehensive survivorship care policies that integrate both cancer surveillance and chronic disease management.

## Simple Summary

Cancer survivors often face ongoing health challenges even after successful treatment, including managing other chronic conditions. Using national survey data collected between 2013 and 2021, we compared health behaviors, general medical and cancer screening rates, and care for high blood pressure, diabetes, and high cholesterol between 2597 cancer survivors and 2458 people without a cancer history. We found that survivors more than five years after diagnosis were attentive to general health check-ups but were less likely to undergo recommended cancer screenings. Additionally, survivors reported lower consistency in managing conditions like high blood pressure and high cholesterol, while engaging in less smoking and drinking. These insights highlight the need for integrated, long-term care plans that support both chronic disease management and cancer-specific screening for cancer survivors.

## 1. Introduction

Cancer is a major public health problem affecting millions of people worldwide [1,2]. Due to advances in treatment and early diagnosis, many people are surviving cancer [3,4]. However, health care for these survivors does not terminate as treatment ends [5,6]. Many experience ongoing health problems after treatment, which can affect their quality of life and long-term health [7,8]. Above all, cancer survivors may experience physical and emotional burdens as a consequence of their cancer treatment, especially concerns about their long-term health following treatment [6,9,10]. This health burden is expressed in various ways, including cancer survivors’ proactive engagement in screenings to maintain their health [11,12,13]. In addition, long-term side effects such as chemotherapy-induced cardiotoxicity or endocrine disorders, and coexisting chronic illnesses including hypertension, diabetes, and dyslipidemia, are prevalent and often exacerbated by unhealthy behaviors such as smoking, inactivity, or poor diet [14,15,16,17,18]. These chronic conditions could potentially worsen the overall health of cancer survivors and may impact their long-term survival outcomes [19,20]. Consequently, these factors increase vulnerability to cardiovascular and metabolic diseases, highlighting the necessity of sustained engagement in preventive care for cancer survivors.

Moreover, cancer survivors often struggle with the management of such chronic diseases [14,18]. Beyond the serious impacts that cancer and its treatment can have on an individual’s daily life and health, managing chronic diseases requires complex and persistent efforts [21,22]. These demands often impose additional burdens on cancer survivors, potentially negatively affecting their health behaviors and outcomes [5,18,23,24]. Numerous studies have indicated that although cancer survivors are generally well aware of the necessity for regular health management due to their cancer history, their adherence to certain general health screenings is paradoxically lower compared to non-survivors. For instance, participation rates among cancer survivors have been reported to be lower than the general population for non-cancer-related screenings, such as blood pressure checks, cholesterol tests, and dental examinations. This phenomenon may be attributable to the relative reprioritization of non-cancer preventive services during the transition back to normal life after treatment [25,26].

Conversely, cancer survivors tend to demonstrate high adherence to cancer-specific screening procedures, such as mammography among breast cancer survivors or colonoscopy among colorectal cancer survivors [27,28]. Nonetheless, compliance with recommended guidelines remains suboptimal within certain demographic groups. This inconsistency is influenced by various factors, including socioeconomic status, accessibility to healthcare services, and physician recommendations, highlighting the necessity for tailored interventions specifically targeting cancer survivors [29,30].

Therefore, this study aims to compare the participation rates and types of regular health screenings and cancer-related screenings between cancer survivors and non-cancer control groups. By identifying screening areas that cancer survivors might relatively neglect, we intend to propose comprehensive long-term follow-up management strategies.

## 2. Materials and Methods

### 2.1. Study Population

The data used in this study were derived from the Korea National Health and Nutrition Examination Survey (KNHANES), an annual cross-sectional survey representative of the national population, conducted under the auspices of the Ministry of Health and Welfare of Korea (MOHW) and the Korea Centers for Disease Control and Prevention (KCDC) [31]. KNHANES participants are subjected to a comprehensive set of questionnaires, encompassing a health interview survey, a health behavior survey, a nutrition survey, and a health screening survey. The participants are selected using a rolling-sample design with a complex, stratified, multistage probability cluster analysis, offering a representative sample of the South Korean population. A three-stage sample design is utilized for the KNHANES. Primary sampling units (PSUs) are drawn from a comprehensive sampling framework that includes all census enumeration areas and residential registration records. Each PSU contains between 50 and 60 residential units on average. Once PSUs have been identified, field teams conduct complete listings of all housing structures within the selected areas, from which 20 households are randomly chosen for screening interviews. The sampling process concludes at the household level, where every resident who is at least one year of age becomes eligible for study participation. As such, the KNHANES is a robust, representative population study with rigorous quality control measures. KNHANES data sets are available through the KCDC website (http://knhanes.cdc.go.kr).

Data for this study were collected between 2013 and 2021. Accordingly, the study period spans 2013–2021, during which all eligible patients meeting the inclusion criteria were observed and analyzed. Participants were identified as cancer survivors if they reported a physician-diagnosed cancer. Given that the KNHANES provided data on both current age and age at the time of cancer diagnosis, we could calculate the duration since the diagnosis of cancer for the study participants. In total, 69,776 unweighted individuals were part of the KNHANES, from which data of 2597 cancer survivor participants were considered. After a 1:1 propensity score matching (PSM) procedure, data from a cumulative 5055 participants, comprising 2597 cancer survivors and 2458 non-cancer controls, were analyzed. Ethical approval was not required for this study because the KNHANES is implemented by an institutional review board of the KCDC (2013-07CON-03-4C), and as mentioned above, the KNHANES data sets, including the one used in this study, are publicly available.

### 2.2. Measures

The sociodemographic characteristics collected in this study are as follows: age (20–39, 40–59, 60–79, and 80 years and above), gender, education level (below middle school graduation, middle school graduation, high school graduation, and bachelor’s degree or higher), marital status (single, married, and widowed/divorced/separated), household income (lower, lower-middle, upper-middle, and upper), and the region of residence (urban and rural). Health-related characteristics included self-reported health status (on a scale of 5, with 5 indicating excellent and 1 indicating very poor), smoking status, and alcohol consumption status.

For cancer diagnosis, the age at which participants were diagnosed with cancer by a physician was recorded. The study also accounted for general health checkups in the past 2 years (“Have you had a general health checkup excluding cancer screening in the past 2 years?”) and cancer screenings (“In the past 2 years, have you ever had a cancer screening?”) conducted within the same timeframe (in the past 2 years). To investigate chronic disease management behaviors, physician-diagnosed hypertension, diabetes, and hyperlipidemia, the three most prevalent chronic diseases in Korea, and the patients’ associated medication adherence were evaluated. Medication adherence was assessed via self-report. We defined ‘low adherence’ as taking the medication on <15 days per month, per the survey’s answer options. In contrast, patients who reported taking their medication ≥15 days per month (including daily use) were categorized as having higher adherence.

### 2.3. Propensity Score Matching (PSM)

Given the potential loss of balance between the two groups, a PSM approach was used to reduce the imbalance of confounding factors between cancer survivors and participants who had never received a cancer diagnosis (controls). Propensity scores were calculated for each group, the group of 2597 cancer survivors and the group of 67,179 controls, with no missing values, through multivariate logistic regression analysis with the demographic characteristics listed in Table 1 as covariates. After estimating propensity scores, one-to-one matching was performed. Participants in the control group were then matched with participants in the cancer survivor group on the basis of closest propensity scores (nearest-neighbor matching with a caliper of 0.2), yielding well-balanced matched groups. The imbalance before and after matching was estimated as an absolute standardized difference, and an absolute standardized difference of less than 10% was considered a non-significant difference.

### 2.4. Statistical Analysis

All statistical analyses were performed using the complex samples procedure as the KNHANES data set was selected through representative, stratified, and cluster sampling methods rather than random sampling methods. Descriptive statistics and Pearson’s chi-square test were used to investigate sociodemographic characteristics and differences between cancer survivors and controls. After PSM, we performed a multivariable logistic regression to identify factors associated with screening participation and medication adherence. We adjusted for potential confounders, including age and sex (model 1). In model 2, sex, age, household income, educational status, marital status, and region are used as adjustment variables. Statistical significance was assumed at *p* < 0.05. All statistical analyses were performed using R software version 4.4.0 (R Core Team, 2024; R Foundation for Statistical Computing, Vienna, Austria).

## 3. Results

### 3.1. Characteristics of Study Groups

The sociodemographic and health-related characteristics of the cancer survivor and control groups are described in Table 1 (before matching) and Table 2 (after 1:1 PSM). There were significant differences in age, gender, household income, education level, marital status, and the region of residence between the two groups before PSM (Table 1). However, after PSM, there was no significant difference between the two groups (Table 2).

### 3.2. Comparison of Screening Rates by a Logistic Regression Analysis After Propensity Matching

As shown in Table 3, there was no difference in the rate of general health examinations between the cancer survivor and control groups. However, in terms of cancer screening, patients with more than 5 years since diagnosis tended to receive significantly less cancer screening than the control group who had never been diagnosed with cancer (odds ratio [OR] = 0.81, *p* = 0.008). These results appeared to be the same when gender and age were adjusted and after all demographic characteristics were adjusted. Therefore, it was confirmed that they received less screening for other types of cancer screening, such as the national cancer screening, as asked in the question.

### 3.3. Comparison of Compliance with Treatment of Chronic Disease by a Logistic Regression Analysis After Propensity Matching

As shown in Table 4, there was no significant difference in the diagnosis rates of hypertension, diabetes, and hyperlipidemia between the cancer survivor group and the control group. However, in terms of adherence to treatment after a diagnosis of hypertension, non-adherence to treatment was significantly higher in cancer survivors with more than 5 years since cancer diagnosis (OR = 1.96, *p* = 0.001). Regarding diabetes, there was no significant difference in the treatment non-adherence rate between the groups. Meanwhile, the non-compliance to treatment after a diagnosis of hyperlipidemia was significantly higher in cancer survivors with more than 5 years since cancer diagnosis (OR = 1.46, *p* = 0.015).

## 4. Discussion

This study aimed to investigate the health behaviors and chronic disease management of cancer survivors by analyzing Korea’s representative data, the KNHANES. Our findings showed that cancer survivors tended to receive less general cancer screening and show less adherence to treatment for chronic diseases, such as hypertension and hyperlipidemia. This pattern was more pronounced among survivors more than 5 years after diagnosis. The result that such non-compliance with health management is observed in a survivor group that is considered completely cured of cancer should be considered important for the survival of cancer survivors.

Contrary to what might have been expected, cancer survivors had lower rates of cancer screening than individuals who had never been diagnosed with cancer. This is contrary to the results of previous studies that cancer survivors attentively receive screening for secondary cancer [5,32]. Furthermore, the study found that cancer survivors with more than 5 years since their diagnosis had a significantly lower rate of cancer screening. Given that cancer survivors are known to be at increased risk of developing secondary malignancies [33,34], these findings underscore the importance of patient education and awareness about the necessity of regular cancer screening, which is crucial even for those who have previously survived a cancer diagnosis.

Unlike previous studies, this study did not show a significant difference in the rate of diagnosis of chronic disease between cancer survivors and non-cancer controls [14,19,35,36]. The results of this study also revealed that adherence to treatment for hypertension and hyperlipidemia in cancer survivors was lower than in those without a history of cancer. Although the treatment compliance of cancer survivors in this study was significantly lower compared to the control group [37,38], the medication compliance shown in this study was higher than in previous studies [37,39,40]. However, significantly lower treatment compliance could be a major threat to the health of cancer survivors who have survived more than 5 years since diagnosis [41,42,43]. One possible interpretation of this finding is that cancer survivors may re-prioritize health concerns after overcoming cancer, focusing more on monitoring cancer recurrence while inadvertently ignoring other chronic disease management [44,45]. The cumulative physical, emotional, and financial burden of cancer treatment may have caused other aspects of their health to be overlooked. However, cardiovascular disease is a leading cause of death in cancer survivors, who generally have more cardiovascular risk factors, such as obesity, hypertension, and diabetes, than the general population [46,47]. This indicates a need to better understand the factors influencing poor medication adherence.

In our study, the cancer survivor group tended to smoke less than the control group. This is similar to previous studies that found that cancer survivors smoked less than individuals with no history of cancer [48,49]. Similarly, the cancer survivor group tended to drink less than the control group in our study, which is again similar to a previous study [50]. This was especially noticeable in cancer survivors with more than 5 years since diagnosis. Although prior research has demonstrated that light drinking can reduce the risk of cardiovascular outcomes [51], high alcohol consumption in cancer survivors may also increase the likelihood of a poor prognosis. Therefore, it is necessary to continuously educate cancer survivors about health behaviors, such as sobriety.

The implications of these findings for healthcare providers and policymakers are profound. Our study underscores the need for a comprehensive long-term care strategy for cancer survivors. This strategy should involve surveillance for potential cancer recurrence and the robust management of other chronic conditions. Our findings emphasize that while a cancer diagnosis and subsequent survival understandably become a focal point for patients and physicians, other health matters, such as chronic disease management, should not be relegated to the sidelines.

This study had several limitations. First, due to the cross-sectional design of the survey, it was not possible to establish a causal relationship between cancer survivorship and health behaviors. Additionally, our findings were based on self-reported data, which may be prone to recall bias. Finally, the inability of our analysis to control for variables, such as the stage of cancer, could influence health behaviors and may have introduced bias into our results.

## 5. Conclusions

Our study indicates that cancer survivors may be falling short not only in managing chronic diseases but also in undergoing regular cancer screenings. There is a clear need for improved patient education and a more comprehensive support system for this population. An emphasis on a holistic, long-term care approach, encompassing both surveillance for potential cancer recurrence and chronic disease management, is paramount. Future research should aim to uncover the reasons behind these disparities and develop targeted interventions to improve the quality of life and overall health outcomes for cancer survivors.

## Figures and Tables

**Table 1:** Characteristics of study participants according to their cancer history ^1^.

		Total	No History of Cancer	Cancer Survivor	*p*-Value
		56,114 (100.0)	53,517 (95.4)	2597 (4.6)	
Gender	Male	24,736 (44.1)	23,723 (44.3)	1013 (39.0)	<0.001
	Female	31,378 (55.9)	29,794 (55.7)	1584 (61.0)	
Age, years	20–39	15,483 (27.6)	15,357 (28.7)	126 (4.9)	<0.001
	40–59	20,688 (36.9)	19,841 (37.1)	847 (32.6)	
	60–79	17,205 (30.7)	15,797 (30.0)	1408 (54.2)	
	≥80	2738 (4.9)	2522 (4.7)	216 (8.3)	
Household income	Lower	10,970 (19.7)	10,221 (19.2)	749 (28.9)	<0.001
	Lower-middle	13,779 (24.7)	13,104 (24.6)	675 (26.1)	
	Upper-middle	15,001 (26.9)	14,395 (27.0)	606 (23.4)	
	Upper	16,065 (28.8)	15,505 (29.1)	560 (21.6)	
Educational status	Below middle school graduation	10,368 (20.9)	9522 (20.2)	846 (32.8)	<0.001
	Middle school graduation	5043 (10.2)	4686 (10.0)	357 (13.8)	
	High school graduation	16,511 (33.3)	15,761 (33.0)	750 (29.1)	
	Bachelor’s degree or higher	17,735 (35.7)	17,107 (36.3)	628 (24.3)	
Marital status	Single	9696 (17.3)	9618 (18.0)	78 (3.0)	<0.001
	Married	38,311 (68.4)	36,340 (68.0)	1971 (75.9)	
	Divorced/Separated/Widowed	8045 (14.4)	7498 (14.0)	547 (21.1)	
Region	Urban	44,980 (80.2)	42,977 (80.3)	2003 (77.1)	<0.001
	Rural	11,134 (19.8)	10,540 (19.7)	594 (22.9)	
Self-reported health status	Excellent	2338 (4.7)	2279 (4.8)	59 (2.3)	<0.001
	Good	12,403 (24.8)	11,980 (25.3)	423 (16.3)	
	Fair	25,470 (51.0)	24,238 (51.2)	1232 (47.5)	
	Poor	7697 (15.4)	7065 (14.9)	632 (24.4)	
	Very poor	2057 (4.1)	1807 (3.8)	250 (9.6)	
Smoking	Never a smoker	35,509 (63.3)	33,865 (63.3)	1644 (63.3)	<0.001
	Current smoker	9339 (16.6)	9132 (17.1)	207 (8.0)	
	Ex-smoker	11,266 (20.1)	10,520 (19.7)	746 (28.7)	
Alcohol	Alcohol consumer	27,363 (51.7)	26,463 (52.5)	900 (34.7)	<0.001
	Non-alcohol consumer	25,614 (48.3)	23,917 (47.5)	1697 (65.3)	

^1^ There are variables not reported by some respondents. As seen in Table 2, for self-reported health status, the control group was more likely to respond with “excellent” or “good.” When cancer survivors were divided into more than 5 years and less than 5 years since diagnosis, there was no significant difference. In terms of smoking, significantly fewer cancer survivors were current smokers, and significantly more were ex-smokers, indicating that this group contained more people who had quit smoking. A similar trend was shown for drinking. Non-drinkers who had not been drinking within 1 month were 65.3% of the cancer survivor group and 56.2% of the control group, showing a tendency for cancer survivors to drink significantly less than those with no history of cancer.

**Table 2:** Characteristics of study participants according to their cancer history after 1:1 propensity score matching.

		Total	No History of Cancer	Cancer Survivor	*p*-Value	Cancer Survivor	Total
						<5 Years	≥5 Years
		5055 (100.0)	2458 (48.6)	2597 (51.4)		1593 (31.5)	1004 (19.9)
Gender	Male	1962 (38.8)	949 (38.6)	1013 (39.0)	N/S	573 (36.0)	440 (43.8)
	Female	3093 (61.2)	1509 (61.4)	1584 (61.0)		1020 (64.0)	564 (56.2)
Age, years	20–39	243 (4.8)	117 (4.8)	126 (4.9)	N/S	58 (3.6)	68 (6.8)
	40–59	1649 (32.6)	802 (32.6)	847 (32.6)		495 (31.1)	352 (35.1)
	60–79	2744 (54.3)	1336 (54.4)	1408 (54.2)		908 (57.0)	500 (49.8)
	≥80	419 (8.3)	203 (8.3)	216 (8.3)		132 (8.3)	84 (8.4)
Household income	Lower	1463 (29.1)	714 (29.2)	749 (28.9)	N/S	493 (31.1)	256 (25.6)
	Lower-middle	1301 (25.8)	626 (25.6)	675 (26.1)		407 (25.6)	268 (26.8)
	Upper-middle	1125 (22.4)	519 (21.0)	606 (23.4)		368 (23.2)	238 (23.8)
	Upper	1145 (22.8)	585 (23.9)	560 (21.6)		320 (20.2)	240 (24.0)
Educational status	Below middle school graduation	1652 (33.8)	806 (35.0)	846 (32.8)	N/S	566 (35.8)	280 (28.1)
	Middle school graduation	704 (14.4)	347 (15.1)	357 (13.8)		206 (13.0)	151 (15.1)
	High school graduation	1398 (28.6)	648 (28.1)	750 (29.1)		456 (28.8)	294 (29.5)
	Bachelor’s degree or higher	1132 (23.2)	504 (21.9)	628 (24.3)		355 (22.4)	273 (27.4)
Marital status	Single	161 (3.2)	83 (3.4)	78 (3.0)	N/S	34 (2.1)	44 (4.4)
	Married	3814 (75.5)	1843 (75.0)	1971 (75.9)		1198 (75.2)	773 (77.1)
	Divorced/Separated/Widowed	1077 (21.3)	530 (21.6)	547 (21.1)		361 (22.7)	186 (18.5)
Region	Urban	3891 (77.0)	1888 (76.8)	2003 (77.1)	N/S	1213 (76.2)	790 (78.7)
	Rural	1164 (23.0)	570 (23.2)	594 (22.9)		380 (23.9)	214 (21.3)
Self-reported health status	Excellent	170 (3.5)	111 (4.8)	59 (2.3)	<0.001	34 (2.1)	25 (2.5)
	Good	922 (18.7)	499 (21.5)	423 (16.3)		278 (17.5)	145 (14.4)
	Fair	2424 (49.3)	1192 (51.3)	1232 (47.5)		748 (47.0)	484 (48.2)
	Poor	1030 (20.9)	398 (17.1)	632 (24.4)		380 (23.9)	252 (25.1)
	Very poor	376 (7.6)	126 (5.4)	250 (9.6)		152 (9.6)	98 (9.8)
Smoking	Never a smoker	3259 (64.5)	1615 (65.7)	1644 (63.3)	<0.001	1036 (65.0)	608 (60.6)
	Current smoker	496 (9.8)	289 (11.8)	207 (8.0)		129 (8.1)	78 (7.8)
	Ex-smoker	1300 (25.7)	554 (22.5)	746 (28.7)		428 (26.9)	318 (31.7)
Alcohol	Alcohol consumer	1976 (39.1)	1076 (43.8)	900 (34.7)	<0.001	590 (37.0)	310 (30.9)
	Non-alcohol consumer	3079 (60.9)	1382 (56.2)	1697 (65.3)		1003 (63.0)	694 (69.1)

N/S = Not significant.

**Table 3:** Comparison of screening rates by a logistic regression analysis after propensity matching.

		Total	Received Screening	Crude		Model 1 ^1^		Model 2 ^2^	
			N (%)	OR	*p*-Value	Adjusted OR	*p*-Value	Adjusted OR	*p*-Value
*Received general medical screening*									
No history of cancer		2316 (47.2)	1674 (72.3)	1.00		1.00		1.00	
Cancer survivor	<5 years	1587 (32.4)	1111 (70.0)	0.90 (0.78–1.03)	0.123	0.90 (0.78–1.04)	0.151	0.94 (0.81–1.09)	0.413
	≥5 years	1003 (20.4)	732 (73.0)	1.04 (0.88–1.22)	0.678	1.02 (0.87–1.21)	0.781	1.05 (0.88–1.24)	0.598
*Received cancer screening*									
No history of cancer		2319 (47.2)	1569 (67.7)	1.00		1.00		1.00	
Cancer survivor	<5 years	1589 (32.4)	1082 (68.1)	1.02 (0.89–1.17)	0.775	1.02 (0.89–1.17)	0.750	1.05 (0.92–1.21)	0.463
	≥5 years	1003 (20.4)	631 (62.9)	0.81 (0.69–0.95)	0.008	0.81 (0.69–0.95)	0.008	0.82 (0.70–0.96)	0.014

^1^ Model 1: Gender and age are used as adjustment variables. ^2^ Model 2: Gender, age, household income, educational status, marital status, and region are used as adjustment variables. N: number. OR: odds ratio.

**Table 4:** Comparison of compliance with treatment of chronic disease by a logistic regression analysis after propensity matching.

		Total	Diagnosis Rate		Non-Adherence to Treatment				Model 1 ^1^		Model 2 ^2^	
			N (%)	*p*-Value	N (%)	*p*-Value	OR	*p*-Value	Adjusted OR	*p*-Value	Adjusted OR	*p*-Value
*Hypertension*												
No history of cancer		2458 (48.6)	964 (39.2)	N/S	69 (7.2)	<0.001	1.00		1.00		1.00	
Cancer survivor	<5 years	1593 (31.5)	580 (36.4)		37 (6.4)		0.88 (0.58–1.34)	0.558	0.90 (0.59–1.36)	0.614	0.95 (0.61–1.45)	0.797
	≥5 years	1004 (19.9)	366 (36.5)		48 (13.1)		1.96 (1.33–2.89)	0.001	1.83 (1.23–2.71)	0.003	1.84 (1.22–2.78)	0.004
*Diabetes*												
No history of cancer		2458 (48.6)	393 (16.0)	N/S	20 (5.1)	N/S	1.00		1.00		1.00	
Cancer survivor	<5 years	1593 (31.5)	264 (16.6)		21 (8.0)		1.61 (0.86–3.04)	0.140	1.64 (0.87–3.11)	0.129	1.57 (0.81–3.05)	0.185
	≥5 years	1004 (19.9)	159 (15.8)		14 (8.8)		1.80 (0.89–3.66)	0.104	1.66 (0.81–3.41)	0.166	1.67 (0.79–3.53)	0.183
*Dyslipidemia*												
No history of cancer		2458 (48.6)	678 (27.6)	N/S	174 (25.7)	0.040	1.00		1.00		1.00	
Cancer survivor	<5 years	1593 (31.5)	448 (28.1)		118 (26.3)		1.04 (0.79–1.36)	0.800	1.03 (0.78–1.36)	0.825	1.03 (0.78–1.35)	0.848
	≥5 years	1004 (19.9)	268 (26.7)		90 (33.6)		1.46 (1.08–1.99)	0.015	1.40 (1.03–1.91)	0.032	1.42 (1.04–1.93)	0.026

^1^ Model 1: Gender and age are used as adjustment variables. ^2^ Model 2: Gender, age, household income, educational status, marital status, and region are used as adjustment variables. N: number. OR: odds ratio.

## Data Availability

KNHANES data sets are available through the KCDC website (http://knhanes.cdc.go.kr).

## Abbreviations

The following abbreviations are used in this manuscript:

KCDC	Korea Centers for Disease Control and Prevention
KNHANES	Korea National Health and Nutrition Examination Study
MOHW	Ministry of Health and Welfare of Korea
OR	Odds ratio

## References

[1] Pilleron S., Soto-Perez-de-Celis E., Vignat J., Ferlay J., Soerjomataram I., Bray F., Sarfati D. (2021). Estimated global cancer incidence in the oldest adults in 2018 and projections to 2050. Int. J. Cancer.

[2] Soerjomataram I., Bray F. (2021). Planning for tomorrow: Global cancer incidence and the role of prevention 2020–2070. Nat. Rev. Clin. Oncol..

[3] Crosby D., Bhatia S., Brindle K.M., Coussens L.M., Dive C., Emberton M., Esener S., Fitzgerald R.C., Gambhir S.S., Kuhn P. (2022). Early detection of cancer. Science.

[4] Siegel R.L., Miller K.D., Fuchs H.E., Jemal A. (2022). Cancer statistics, 2022. CA Cancer J. Clin..

[5] Kenzik K.M. (2019). Health care use during cancer survivorship: Review of 5 years of evidence. Cancer.

[6] Miroševič Š., Prins J.B., Selič P., Zaletel Kragelj L., Klemenc Ketiš Z. (2019). Prevalence and factors associated with unmet needs in post-treatment cancer survivors: A systematic review. Eur. J. Cancer Care.

[7] Niedzwiedz C.L., Knifton L., Robb K.A., Katikireddi S.V., Smith D.J. (2019). Depression and anxiety among people living with and beyond cancer: A growing clinical and research priority. BMC Cancer.

[8] Lovelace D.L., McDaniel L.R., Golden D.J. (2019). Long-term effects of breast cancer surgery, treatment, and survivor care. J. Midwifery Women’s Health.

[9] Hendriks M.J., Harju E., Michel G. (2021). The unmet needs of childhood cancer survivors in long-term follow-up care: A qualitative study. Psycho-Oncology.

[10] Rutherford C., Müller F., Faiz N., King M.T., White K. (2020). Patient-reported outcomes and experiences from the perspective of colorectal cancer survivors: Meta-synthesis of qualitative studies. J. Patient-Rep. Outcomes.

[11] Shin D.W., Kim Y.W., Oh J.H., Kim S.W., Chung K.W., Lee W.Y., Lee J.E., Lee W.C., Guallar E., Cho J. (2011). Knowledge, attitudes, risk perception, and cancer screening behaviors among cancer survivors. Cancer.

[12] Millar M.M., Edwards S.L., Herget K.A., Orleans B., Ofori-Atta B.S., Kirchhoff A.C., Carter M.E., Nagata M., Sweeney C. (2023). Adherence to Guideline-Recommended cancer screening among Utah cancer survivors. Cancer Med..

[13] Simard S., Thewes B., Humphris G., Dixon M., Hayden C., Mireskandari S., Ozakinci G.J. (2013). Fear of cancer recurrence in adult cancer survivors: A systematic review of quantitative studies. J. Cancer Surviv..

[14] Renzi C., Kaushal A., Emery J., Hamilton W., Neal R.D., Rachet B., Rubin G., Singh H., Walter F.M., de Wit N.J. (2019). Comorbid chronic diseases and cancer diagnosis: Disease-specific effects and underlying mechanisms. Nat. Rev. Clin. Oncol..

[15] Corbett T., Cummings A., Calman L., Farrington N., Fenerty V., Foster C., Richardson A., Wiseman T., Bridges J. (2020). Self-management in older people living with cancer and multi-morbidity: A systematic review and synthesis of qualitative studies. Psycho-Oncology.

[16] Lee S.J., Cartmell K.B. (2021). An association rule mining analysis of lifestyle behavioral risk factors in cancer survivors with high cardiovascular disease risk. J. Pers. Med..

[17] Armenian S.H., Xu L., Ky B., Sun C., Farol L.T., Pal S.K., Douglas P.S., Bhatia S., Chao C. (2016). Cardiovascular disease among survivors of adult-onset cancer: A community-based retrospective cohort study. J. Clin. Oncol..

[18] Howell D., Mayer D.K., Fielding R., Eicher M., Verdonck-de Leeuw I.M., Johansen C., Soto-Perez-de-Celis E., Foster C., Chan R., Alfano C.M. (2021). Management of cancer and health after the clinic visit: A call to action for self-management in cancer care. J. Natl. Cancer Inst..

[19] Islam K.M., Jiang X., Anggondowati T., Lin G., Ganti A.K. (2015). Comorbidity and survival in lung cancer patients. Cancer Epidemiol. Biomark. Prev..

[20] Søgaard M., Thomsen R.W., Bossen K.S., Sørensen H.T., Nørgaard M. (2013). The impact of comorbidity on cancer survival: A review. Clin. Epidemiol..

[21] Lagergren P., Schandl A., Aaronson N.K., Adami H.O., de Lorenzo F., Denis L., Faithfull S., Liu L., Meunier F., Ulrich C. (2019). Cancer survivorship: An integral part of Europe’s research agenda. Mol. Oncol..

[22] Mayer D.K., Alfano C.M. (2019). Personalized risk-stratified cancer follow-up care: Its potential for healthier survivors, happier clinicians, and lower costs. J. Natl. Cancer Inst..

[23] Bowers A., Karvetski C.H., Needs P. (2022). Cost Burden Impacts Cancer Patient Service Utilization Behavior in a US Commercial Plan. Am. J. Health Behav..

[24] Nipp R.D., Kirchhoff A.C., Fair D., Rabin J., Hyland K.A., Kuhlthau K., Perez G.K., Robison L.L., Armstrong G.T., Nathan P.C. (2017). Financial burden in survivors of childhood cancer: A report from the Childhood Cancer Survivor Study. J. Clin. Oncol..

[25] Avila J.C., Kuo Y.-F., Rodriguez A.M., Wong R., Kaul S. (2017). Preventive services use among female survivors of adolescent and young adult cancer. J. Cancer Surviv..

[26] Mandelzweig L., Chetrit A., Amitai T., Silverman B., Siegelmann-Danieli N., Sadetzki S. (2017). Primary prevention and screening practices among long-term breast cancer survivors. Cancer Causes Control.

[27] Mayer D.K., Terrin N.C., Menon U., Kreps G.L., McCance K., Parsons S.K., Mooney K.H. (2007). Screening practices in cancer survivors. J. Cancer Surviv..

[28] Mesa-Chavez F., Salazar-Alejo M., Villarreal-Garza C. (2024). Screening Adherence for Second Primary Malignancies in Breast Cancer Survivors: Behaviors, Facilitators, and Barriers to Enhance Quality Care. Semin. Oncol..

[29] Beatrici E., Qian Z., Filipas D.K., Stone B.V., Dagnino F., Labban M., Lipsitz S.R., Lughezzani G., Buffi N.M., Cole A.P. (2024). Socio-Economic Determinants of Cancer Screening Adherence Among Cancer Survivors: Analysis from 2020 Behavioral Risk Factor Surveillance System. JNCI Cancer Spectr..

[30] MacDonald M., Mirza A.-S., Mhaskar R., Ewing A., Chen L., Robinson K., Lu Y., Ayoubi N., Gonzalez E., Guerra L. (2022). Preventative Cancer Screening Rates Among Uninsured Patients in Free Clinics: A Retrospective Cohort Study of Cancer Survivors and Non-cancer Survivors. Cancer Control.

[31] Oh K., Kim Y., Kweon S., Kim S., Yun S., Park S., Lee Y.-K., Kim Y., Park O., Jeong E.K. (2021). Korea National Health and Nutrition Examination Survey, 20th anniversary: Accomplishments and future directions. Epidemiol. Health.

[32] Corkum M., Hayden J.A., Kephart G., Urquhart R., Schlievert C., Porter G. (2013). Screening for new primary cancers in cancer survivors compared to non-cancer controls: A systematic review and meta-analysis. J. Cancer Surviv..

[33] Fidler M.M., Frobisher C., Hawkins M.M., Nathan P.C. (2019). Challenges and opportunities in the care of survivors of adolescent and young adult cancers. Pediatr. Blood Cancer.

[34] Salloum R., Chen Y., Yasui Y., Packer R., Leisenring W., Wells E., King A., Howell R., Gibson T.M., Krull K.R. (2019). Late morbidity and mortality among medulloblastoma survivors diagnosed across three decades: A report from the childhood cancer survivor study. J. Clin. Oncol..

[35] Deckx L., van den Akker M., Metsemakers J., Knottnerus A., Schellevis F., Buntinx F. (2012). Chronic diseases among older cancer survivors. J. Cancer Epidemiol..

[36] Bullard T., Ji M., An R., Trinh L., Mackenzie M., Mullen S.P. (2019). A systematic review and meta-analysis of adherence to physical activity interventions among three chronic conditions: Cancer, cardiovascular disease, and diabetes. BMC Public Health.

[37] Yang J., Neugut A.I., Wright J.D., Accordino M., Hershman D.L. (2016). Nonadherence to oral medications for chronic conditions in breast cancer survivors. J. Oncol. Pract..

[38] Neugut A.I., Zhong X., Wright J.D., Accordino M., Yang J., Hershman D.L. (2016). Nonadherence to Medications for Chronic Conditions and Nonadherence to Adjuvant Hormonal Therapy in Women with Breast Cancer. JAMA Oncol..

[39] Calip G.S., Elmore J.G., Boudreau D.M. (2017). Characteristics associated with nonadherence to medications for hypertension, diabetes, and dyslipidemia among breast cancer survivors. Breast Cancer Res. Treat..

[40] Trogdon J.G., Amin K., Gupta P., Urick B.Y., Reeder-Hayes K.E., Farley J.F., Wheeler S.B., Spees L., Lund J.L. (2021). Providers’ mediating role for medication adherence among cancer survivors. PLoS ONE.

[41] Ko A., Kim K., Sik Son J., Park H.Y., Park S.M. (2019). Association of pre-existing depression with all-cause, cancer-related, and noncancer-related mortality among 5-year cancer survivors: A population-based cohort study. Sci. Rep..

[42] Tollosa D.N., Holliday E., Hure A., Tavener M., James E.L. (2020). A 15-year follow-up study on long-term adherence to health behaviour recommendations in women diagnosed with breast cancer. Breast Cancer Res. Treat..

[43] Feng J.L., Qin X. (2021). Does adherence to lipid-lowering medications improve cancer survival? A nationwide study of breast and colorectal cancer, and melanoma. Br. J. Clin. Pharmacol..

[44] Götze H., Taubenheim S., Dietz A., Lordick F., Mehnert-Theuerkauf A. (2019). Fear of cancer recurrence across the survivorship trajectory: Results from a survey of adult long-term cancer survivors. Psycho-Oncology.

[45] Luigjes-Huizer Y.L., Tauber N.M., Humphris G., Kasparian N.A., Lam W.W., Lebel S., Simard S., Smith A.B., Zachariae R., Afiyanti Y. (2022). What is the prevalence of fear of cancer recurrence in cancer survivors and patients? A systematic review and individual participant data meta-analysis. Psycho-Oncol..

[46] Vincent L., Leedy D., Masri S.C., Cheng R.K. (2019). Cardiovascular disease and cancer: Is there increasing overlap?. Curr. Oncol. Rep..

[47] Oh S., Lee J., Hong Y.S., Kim K. (2023). Increased risk of cardiovascular disease associated with diabetes among adult cancer survivors: A population-based matched cohort study. Eur. J. Prev. Cardiol..

[48] Gummerson S.P., Lowe J.T., Taylor K.L., Lobo T., Jensen R.E. (2022). The characteristics of patients who quit smoking in the year following a cancer diagnosis. J. Cancer Surviv..

[49] Grimmett C., Bridgewater J., Steptoe A., Wardle J. (2011). Lifestyle and quality of life in colorectal cancer survivors. Qual. Life Res..

[50] Lin H.-Y., Fisher P., Harris D., Tseng T.-S. (2019). Alcohol intake patterns for cancer and non-cancer individuals: A population study. Transl. Cancer Res..

[51] Chiva-Blanch G., Badimon L. (2019). Benefits and risks of moderate alcohol consumption on cardiovascular disease: Current findings and controversies. Nutrients.

